# Identification of Potential Environmentally Adapted *Campylobacter jejuni* Strain, United Kingdom

**DOI:** 10.3201/eid1411.071678

**Published:** 2008-11

**Authors:** Will Sopwith, Andrew Birtles, Margaret Matthews, Andrew Fox, Steven Gee, Michael Painter, Martyn Regan, Qutub Syed, Eric Bolton

**Affiliations:** Health Protection Agency (North West), Liverpool, UK

**Keywords:** Campylobacter jejuni, isolation, epidemiology, transmission, multilocus sequence typing, children, longitudinal studies, United Kingdom, dispatch

## Abstract

In a study of *Campylobacter* infection in northwestern England*,* 2003–2006, *C. jejuni* multilocus sequence type (ST)–45 was associated with early summer onset and was the most prevalent *C. jejuni* type in surface waters. ST-45 is likely more adapted to survival outside a host, making it a key driver of transmission between livestock, environmental, and human settings.

Human campylobacterosis shows a marked seasonality with a peak during the early summer months in many countries (*1*). The driving factors for this seasonality are not understood. Studies have shown a coincident seasonality of infection in chicken, livestock, and humans, and the possibility of a common environmental trigger has been suggested (*2*). In a recent study of the influence of climate on seasonality in England and Wales, incidence of campylobacteriosis was correlated with air temperature (with higher temperature indicating more cases at key points of the year) (*3*). This finding may relate to animal husbandry practices, especially animal housing (*4*).

Studies have attempted to identify environmental reservoirs of infection in water sources; *Campylobacter* organisms have been successfully cultured from surface water (*5*), and campylobacteriosis has been linked with exposure to untreated water (*6*). We were interested in identifying the factors driving the early summer increase of cases in the United Kingdom and in investigating the role of environmental reservoirs. Preliminary data identified multilocus sequence type (ST)–45 complex as a strain with possible transmission from environmental sources (*7*), and we have analyzed this complex in more detail.

## The Study

The study population was defined as all human cases of laboratory-confirmed *Campylobacter* infection with onset from April 2003 through March 2006, reported by residents in 4 local authorities in northwestern England, as previously described (*7*). All case-patients were asked detailed questions about their illnesses and possible exposures.

Water samples were collected at least each fortnight from October 2003 through December 2005 as 2-L grab samples from sampling points on 2 rivers associated with the study area (River Mersey and River Wyre). Water samples were transported to the Food and Environmental Microbiology Laboratory, Royal Preston Hospital. *Campylobacter* species were isolated by the addition of 10 mL of the water sample to 90 mL of warmed *Campylobacter* enrichment broth (product CM0983, Oxoid Ltd, Basingstoke, UK) and incubated at 37°C for 24 hours, followed by incubation at 42°C for 24 hours. The enrichment broths were subcultured onto *Campylobacter* blood-free selective agar (charcoal cefoperazone deoxycholate agar product CM0739, Oxoid Ltd) at 37°C for 48 hours microaerobically, by using a microaerobic gas generating kit (product CN0025, Oxoid, Ltd). *Campylobacter* colonies were identified by morphologic features and confirmed by microaerobic and aerobic growth on blood agar. The colonies were then placed in Amies transport and sent to the laboratory Health Protection Agency regional laboratory in Manchester, UK, for DNA extraction and characterization. *C. jejuni* isolates were identified to species and typed by multilocus sequence typing as previously described (*7*).

Case–case methodology was used to compare exposures between sequence types of *Campylobacter* (*8*). Statistical analysis was performed by using STATA version 9.2 (StataCorp, College Station, TX, USA). Two-way tabulations and Fisher exact test were used to estimate the direction and size of association of individual variables with sequence types. Logistic regression was performed to estimate the significance of these findings and to examine them in a multivariate model. Data were collected from 2 distinct locations, and, thus, area of residence was controlled for in analysis as a stratified variable. Likelihood ratio tests were used to assess the significance of including or excluding variables from multivariate analysis.

**Figure 1:**
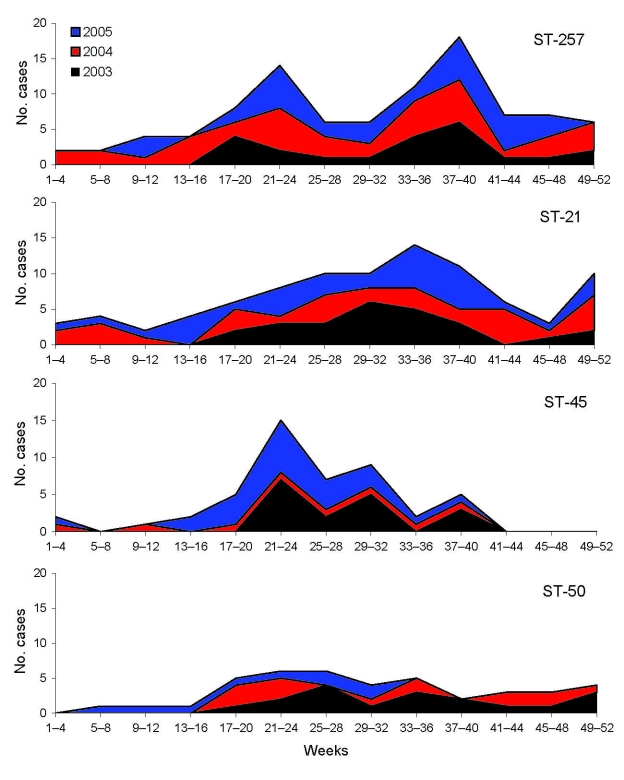
Stacked area charts showing the number of human case reports of the 4 most prevalent sequence types (STs) during the study, by 4-week intervals.

Among the typed isolates of *C*. *jejuni* (n = 1,104), ST-45 (n = 49) was the third most prevalent sequence type reported in the study (after ST-257 and ST-21) and highly restricted to cases reported between late April (week 17) and early August (week 32) (Figure 1). When week of diagnosis was categorized into 2 seasons (early summer [weeks 17–28] and all other weeks), logistic regression of season by sequence type confirmed that, when compared with all other typed cases of *C. jejuni*, cases of ST-45 were more likely to be reported in early summer than during the rest of the year (odds ratio 2.79, confidence interval 1.56–4.99, p = 0.001; 1,008 observations), and this relationship was not seen with other prevalent sequence types.

**Figure 2:**
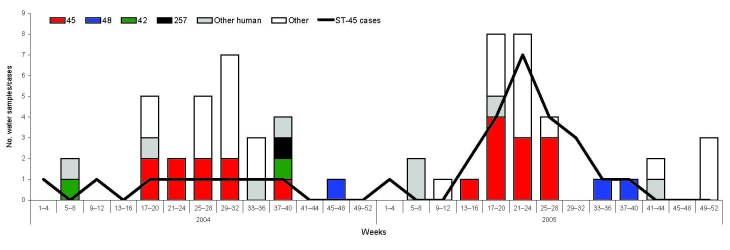
Numbers and sequence types (STs) of isolates of *Campylobacter jejuni* from surface water samples compared with numbers of human cases of ST-45 (line) for 2004 and 2005, by 4-week interval. Only the 4 most prevalent human types also identified in water samples are distinguished (ST-257, ST-45, ST-21, and ST-48). The “Other human” category includes all other *C. jejuni* sequence types found both in human cases in the study and in water samples. The “Other” category includes other *C. jejuni* sequence types found in water samples but not in human case-patients in the study.

Seventy-four (37%) of 198 river water samples were positive for *C. jejuni*, and among these, 28 sequence types (Table 1) were identified, 11 of which were also reported among human cases in the study population. The most prevalent sequence type identified was ST-45, and the seasonality of isolation appears to closely correlate with the seasonality of human disease onset caused by this type (Figure 2).

**Table 1:** Isolates of *Campylobacter jejuni* recovered from river water samples in 2004 and 2005, showing clonal complex and ST*†

Clonal complex/ST	Water isolates	Human cases	Prevalence among human cases, %
ST-45			
45	21	Y	4.83
2405	2		–
2406	1		–
230	1		–
714	1		–
2219	1		–
137	1	Y	1.08
ST-21			
21	2	Y	9.37
53	1	Y	3.55
UA	1		–
ST-48			
48	3	Y	3.85
475	1	Y	1.48
ST-42			
42	2	Y	1.38
1751	1		–
ST-1332			
2404	1		–
696	1		–
ST-508			
2187	2		–
ST-179			
220	1		–
ST-460			
606	1	Y	0.10
ST-403			
415	1		–
ST-658			
UA	1		–
ST-677			
677	1	Y	0.69
ST-257			
257	1	Y	9.76
ST-1388			
177	1		–
ST-61			
61	1	Y	1.38
UA			
UA	4		–
448	2		–
2408	1		–
789	1		–
947	1		–
2407	1		–
Total	61		

*ST, sequence type; UA, unassigned or newly identified sequence type at time of publication; –, blank values. †Those STs also found in human cases of *C. jejuni* among the study population are indicated with a Y and their prevalence indicated as a percentage of all typed cases of *C. jejuni* in the study population.

Univariate logistic regression analysis identified that, in addition to illness during early summer, the statistically strongest associations (p<0.1) with ST-45 were being <5 years of age and living in the more rural part of the study area. These 3 factors remained significantly and independently more associated with ST-45 infection than with other sequence types when combined in a multivariate model (tested by using likelihood ratio tests) (Table 2). When this model was used, subsequent estimation of the association of food and exposure variables showed that consumption of chicken at least once was less associated and consumption of home-delivered milk, and going fishing were more associated with ST-45 than with other types. This finding was sustained in a multivariate model (also including the demographic variables previously mentioned) (Table 2). However, information on exposure variables was not available for all 1,008 cases of *C. jejuni*, which resulted in a loss of statistical power.

**Table 2:** Two-step multivariate logistic regression analysis of epidemiologic variables associated with cases of ST-45, in comparison with all other sequence types of *Campylobacter jejuni* *

Variable	Univariate				Multivariate		
	OR	CI	p value		OR	CI	p value
Initial model (obs = 1,008)†							
Onset during early summer	2.79	1.56–4.99	0.001		2.72	1.52–4.89	0.001
Age <5 y	3.48	1.54–7.86	0.003		3.32	1.45–7.61	0.005
Rural residence	1.96	1.10–3.51	0.023		2.03	1.13–3.66	0.018
Final model (controlled for above)‡					Final multivariate (obs = 580)		
Going fishing in the 2 weeks before illness (obs = 737)	3.29	0.69–15.80	0.137		3.95	0.71–22.0	0.118
Consumption of home-delivered milk (obs = 633)	1.99	0.85–4.64	0.113		2.45	0.97–6.17	0.058
Consumption of chicken at least once (obs = 645)	0.23	0.08–0.66	0.006		0.21	0.07–0.63	0.006

*obs, number of observations for each analysis (i.e., no. cases in the study with available information); OR, odds ratio, CI, confidence interval. †Initial analysis of basic demographics (obs = 1,008) showed being ill in the early summer (weeks 17–28) and being <5 y of age were independently associated with cases of infection with ST-45, when controlled for the stratified variable of residential area. ‡Further analysis of exposures (controlled for the initial model) showed going fishing, consuming chicken, and consuming home-delivered milk to be independently associated with cases of infection with ST-45, and these associations remained in the final multivariate model, including all variables listed in the table. For the full regression model, only 580 of the possible 1,008 cases had information on all the variables of interest.

## Conclusions

We have shown that a single prevalent human strain of *C. jejuni*, ST-45, is strongly associated with the early summer seasonal peak of campylobacteriosis incidence described previously in northwestern England (*7*). This strain is also frequently isolated from recreational surface waters adjacent to the populations studied, from which other prevalent strains have rarely been isolated. The concordance between period of ST-45 isolation in water and reported incidence in humans is striking and suggests a relationship between the presence of this strain in the environment and human infection. When compared with case-patients infected by other sequence types of *C. jejuni*, persons infected with ST-45 are more likely to live in rural areas, to be <5 years of age, to have gone fishing before illness, or to have consumed home-delivered milk, and were much less likely to have eaten any chicken in the 2 weeks before illness.

The coincident seasonal presence of ST-45 in both surface water and in case-patients may arise simply through seasonal excretion of ST-45, resulting from well-characterized seasonal infection in humans (*1*,*3**,**7*) or livestock. The river systems sampled were both urban and rural in character, and contamination through human sewage discharge or animal feces from adjoining pastures is possible. However, other common human and animal *Campylobacter* sequence types were largely absent from the water sampled.

Although ingestion of untreated surface waters has previously been shown to be a risk factor for campylobacteriosis in a UK-wide case-control study (*9*), these new data are not sufficiently robust to demonstrate a causal link for ST-45 infection. Although data were collected on proxy exposures to water, reported exposures were very low. Furthermore, systematic water sampling would be required to confirm the apparent seasonal positivity of ST-45 in these data. The potential role of pets, and in particular dogs, in bridging the gap between exposure to surface water (for example, while being exercised) and domestic exposure settings remains to be investigated. Although cases of infection caused by ST-45 were no more associated with owning dogs than were cases of other sequence types (data not shown), the exposures of those dogs were not recorded. In a recent study, cases of ST-45 clonal complex were more associated with contact with pet dogs and cats than were other clonal complexes identified (*10*); pet-mediated transmission of ST-45 might be supported in this study by the observed association of ST-45 with young children.

Despite the evidence presented of a potential environmental transmission route for ST-45, it is also a type well recognized to colonize poultry (*10*–*12*), and the incidence among humans may be a result of consumption of seasonally contaminated poultry (*2*). However, the absence of strong early summer seasonality among other recognized chicken-adapted sequence types suggests that this is not the case (data not shown). Also, the evidence from our study is strong that consumption of chicken (a common human exposure) is less associated with human ST-45 infection than with other types.

An explanation for these observations may be that ST-45 represents a strain of *Campylobacter* that is comparatively well adapted to survival outside an animal host, as has been hypothesized for some strains of *C. coli* (*13*). Other studies have reported that ST-45 is more widely distributed in terms of host and ecologic niche, including water, than other common sequence types (*12*,*14*). Evidence also has indicated that ST-45 is more resilient to physical stress than other sequence types (*15*). This would certainly support the hypothesis that ST-45 was more available to infect humans through transmission routes other than food, either through direct exposure to water or the countryside through outdoor activities or by indirect exposure through pets. The hypothesis is further supported by the association of human ST-45 with more rural area of residence in these data. The availability of ST-45 to humans due to its hypothesized adaptive survival outside animal hosts would apply even more so to poultry, because of their increased environmental exposure, and 1 study has demonstrated that contamination of a new flock with ST-45 arose from an isolate in a puddle outside a chicken house (*12*). Thus, through an as-yet-uncharacterized adaptation, ST-45 may be a strain of *C. jejuni* that is able to bridge the various recognized environmental, livestock, and human transmission settings for disease, making it a key target for intervention in reducing *Campylobacter* prevalence. It may also be a key driver for the early summer rise in human incidence, both through nonfoodborne exposure and contamination of food animals.
